# Platinum-Based Chemotherapy and Immunotherapy in Early Triple-Negative Breast Cancer: A Meta-Analysis and Indirect Treatment Comparison

**DOI:** 10.3389/fonc.2021.693542

**Published:** 2021-07-01

**Authors:** Qin He, Yicheng Peng, Jie Sun, Jianxia Liu

**Affiliations:** Department of General Surgery, The First Affiliated Hospital of Soochow University, Suzhou, China

**Keywords:** platinum, immune checkpoint inhibitors, triple negative breast cancer, neoadjuvant therapy, meta - analysis, indirect treatment comparison

## Abstract

**Background:**

Triple-negative breast cancer (TNBC) comprises 15% of invasive breast cancers. Platinum-based chemotherapy and immune checkpoint inhibitors (ICIs) have been extensively researched in recent years as promising treatments in the neoadjuvant setting. However, clinical data is lacking in direct comparisons of these two treating regimens.

**Methods:**

We conducted an online search on PubMed, Embase, Cochrane Online Library and key oncological meetings for available randomized controlled trials (RCTs) investigating ICIs or platinum drugs *versus* anthracyclines and taxane-based neoadjuvant chemotherapy (AT-based NACT). Conventional meta-analyses were conducted separately, and then indirect comparisons for clinical efficacy and safety profile were performed between ICIs and platinum drugs using AT-based NACT as a common comparator.

**Results:**

Seven random controlled trials (RCTs) with 1,647 patients were included in the meta-analysis. The indirect comparison demonstrated that ICIs plus chemotherapy significantly improved pathological complete response (pCR) rate (p = 0.00445, OR, 1.78; 95%CI, 0.70–4.53), and decreased the adverse effect (AE) related discontinuance *versus* platinum-based chemotherapy (P = 0.00015; OR 0.46; 95%CI, 0.26–0.82).

**Conclusion:**

ICIs plus chemotherapy showed increased pCR rate and decreased adverse effects compared with platinum-based chemotherapy in early TNBC. However, subgroup analysis and survival data to explore the proper patients for each treatment remains scarce. Therefore, further studies with powered direct comparisons of these two treating regimens are required.

## Introduction

Breast cancer is the most common cancer in women and one of the most common causes of cancer-related deaths worldwide. Triple-negative breast cancer (TNBC) is recognized as the most aggressive subtype that comprises 15% of invasive breast cancers with a higher recurrence rate and poor outcome (1). It is defined as tumors that lack expression of estrogen receptor, progesterone receptor, and human epidermal growth factor receptor 2 (HER2), leading to a shortage of therapeutic targets and posing a treatment challenge (2).

Chemotherapy remains the most widely applied and efficient systemic treatment of TNBC. As the standard treatment option regarding early stage TNBC, neoadjuvant chemotherapy has a higher pathological complete response (pCR) rates compared to hormone receptor positive subtype (3). Additionally, studies have proved that pCR in the neoadjuvant setting is associated with improved long-term outcomes in TNBC (4, 5).

Around 15 to 25% of TNBC patients commonly harbor BRCA gene mutations (6), which make them susceptible to DNA-damaging compounds such as platinum drugs (7). With traditional neoadjuvant chemotherapy, including anthracycline and taxane regimens, the pCR rate of TNBC is about 27.7%, while platinum-based chemotherapy improves it to 40.1% (8). However, many patients appear to be particularly insensitive to chemotherapy due to the molecular heterogeneity of TNBC subtype (9), that requires more clinical research studies to develop targeted therapies and optimizing the therapeutic strategy for the neoadjuvant treatment of TNBC.

Recent studies on immune checkpoint inhibitors (ICIs) have provided a new treatment strategy for TNBC. Effector T-cells express the programmed death 1 (PD-1) cell surface receptor, which interacts with its ligand PD-L1, and leads to the inhibition of cytotoxic T-cells (10). By targeting tumors enriched with tumor-infiltrating lymphocytes (TILs) that express PD-L1, T-cells within the tumor microenvironment can be activated to mediate tumor cell killing (11). TNBC is recognized as the immunogenic subtype with more TILs, higher PD-1 expression, and a higher median tumor mutational burden compared with other breast cancers (12). Therefore, the clinical development of ICI is more advanced in TNBC.

In recent years, several studies have investigated platinum-based chemotherapy and ICIs separately. However, there has not been enough study to directly compare platinum drugs with ICIs. In lieu of head-to-head randomized control trials, we summarized recent and relevant trials and performed an indirect comparison between the two treatments of TNBC.

## Materials and Methods

### Study Design

The main purpose of this study is to compare the efficacy and safety between platinum-based chemotherapy and ICIs indirectly, using standard anthracycline and taxane-based neoadjuvant chemotherapy (AT-based NACT) as a common comparator. The conventional meta-analyses on platinum-based with AT-based NACT and ICIs plus chemotherapy with AT-based NACT were conducted separately. Based on the results of the two meta-analyses, a common reference-based indirect comparison was performed on platinum and ICIs mediated by AT-based NACT.

### Literature Search and Selection Criteria

A literature review was conducted in PubMed, Embase, and the Cochrane library (last updated in February 2021). Annual conference presentations were also searched, including the American Society of Clinical Oncology meetings and San Antonio Breast Cancer Symposium. To identify relevant studies, the following terms were employed as queries: “immune checkpoint inhibitor” or “PD-1” or “PD-L1” or “durvalumab” or “pembrolizumab” or “atezolizumab”, “carboplatin” or “cisplatin” or “platinum”, “neoadjuvant therapy” and “triple-negative breast cancer”. Eligible studies had to meet the following inclusion criteria: (1) randomized controlled trials (RCTs) in patients with early stage breast cancer; (2) enrolling TNBC patients receiving AT-based NACT in the control arm and either carboplatin-based chemotherapy or ICIs with AT-based NACT in the experimental arm; (3) reporting data on pCR after neoadjuvant treatment. Studies excluded were (1) reviews, case reports or non-RCTs; (2) RCTs involving other breast cancer subtypes without separate results on TNBC subgroup; (3) ongoing studies without published results at the time of the literature search; (4) with control arm given chemotherapy other than AT-based NACT.

### Data Extraction

The following information was extracted from each study: name of the trial, year of publication, study population, patient number in both control arm and experimental arm, number of patients achieving pCR (defined as no residual invasive tumor at the time of surgery in both breast and axilla, *i.e.* ypT0/Tis ypN0 or no residual invasive or *in situ* tumor in both breast and axilla, *i.e.* ypT0 ypN0). The event-free survival (EFS, defined as time from randomization to disease recurrence, progression or death because of any cause) was extracted when available. The toxicity profile was also extracted in terms of treatment discontinuations related to adverse effects (AEs) and main grade 3–4 AEs. In trials enrolling patients with different breast cancer subtypes, only data in the TNBC group was included. For investigations with more than one report, data were gathered from the most recent findings.

### Quality Evaluation

The risk of bias of the eligible studies was assessed using the Cochrane Collaboration bias assessment tool for a systematic review of interventions. Selection bias (parameters of details of random sequence generation and allocation concealment), performance bias (blinding for participants and personnel), detection bias (blinding for outcome assessment), attrition bias (incomplete outcome data), reporting bias (selective reporting), and other biases were assessed. The risk of bias was stratified as high, low, or unclear.

### Statistical Analysis

Meta-analysis was conducted using Review Manager software (RevMan, version 5.3 for windows; Cochrane Collaboration, Oxford, UK). The odds ratio (OR) and 95% confidence intervals (CIs) were quantitatively synthesized for the effect on pCR. The between-study heterogeneity was evaluated using the I^2^ test, in which the heterogeneity was designated as I^2^ >50%. In the absence of statistically significant heterogeneity, we calculated the pooled effect using a fixed-effects model. With significant heterogeneity, on the other hand, we employed a random-effects model. Sensitivity analysis was performed to investigate the influence of a single study on the overall incidence using STATA software (version14.0 STATA Corporation, TX, USA). Funnel plots were generated using RevMan to detect publication bias. To preliminarily investigate the difference between platinum and ICIs, due to the fact that a direct comparison study was currently lacking, we made an indirect comparison of the two neoadjuvant therapy using ITC 1.0 (Canadian Agency for Drugs and Technologies in Health, Ontario, Canada).

## Results

### Characteristics of the Studies

**Figure 1:**
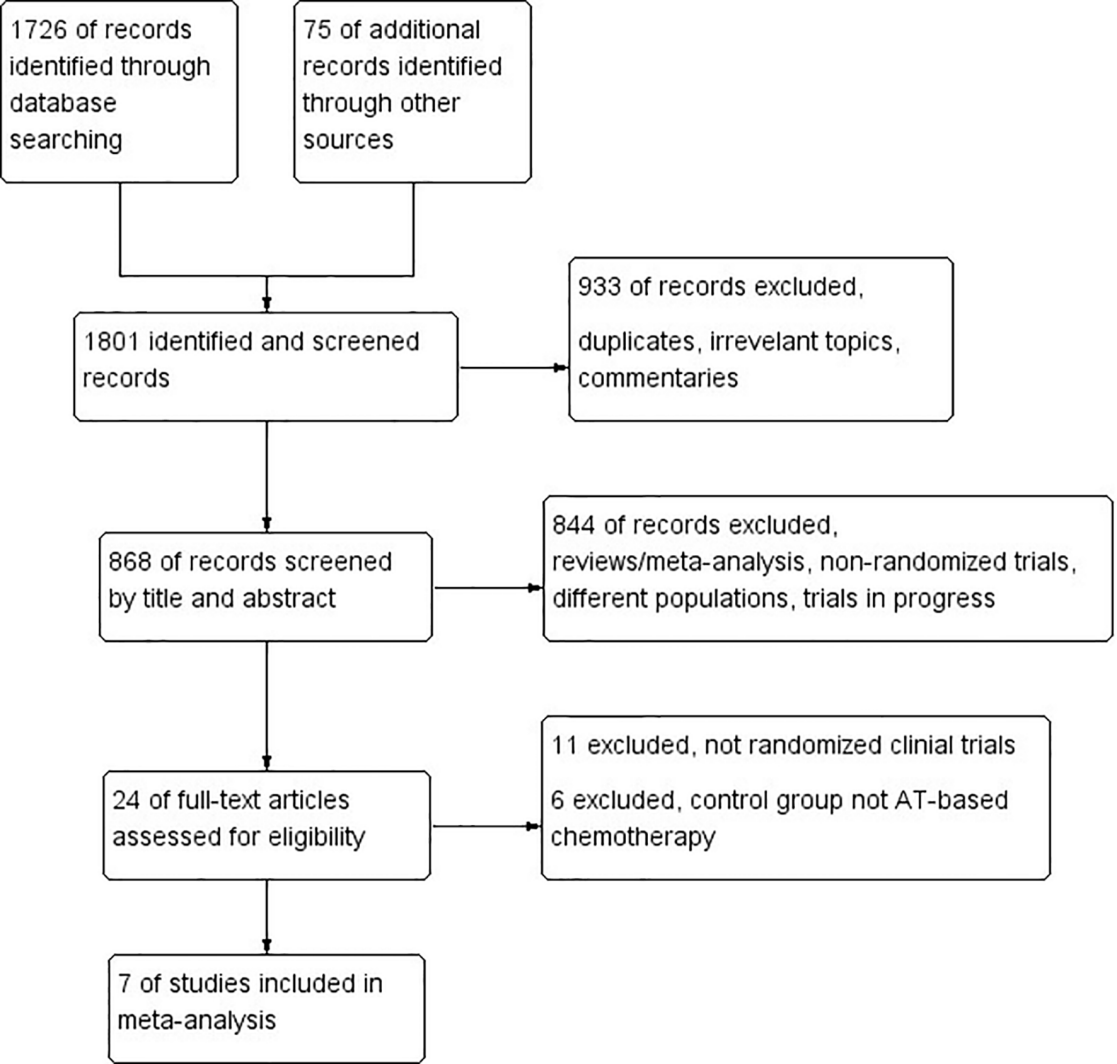
PRISMA flow chart summarizing the process for the identification of eligible randomized controlled trials.

A total of seven RCTs (1–19) fulfilled the eligibility criteria (**Table 1**), among which four trials compared platinum-based with AT-based NACT, while three compared ICIs plus chemotherapy with chemotherapy alone. The search progress is shown in **Figure 1**. Two of the studies included other subtypes of breast cancer, and two of the studies involved multiple treating arms besides AT-based NACT and platinum-based therapy, but only the data pertaining to TNBC subtype or eligible treating arms were extracted in this meta-analysis.

**Table 1:** Characteristics of eligible studies.

Study	Year	Phase	Population	Platinum/ICI group	pCR	AT group	pCR
BrignTNess (13)	2018	3	stage II–III TNBC	PCb-AC	57.50%	P-AC	31.01%
CALGB (14)	2014	2	stage II–III TNBC	PCb-ddAC	48.65%	P-ddAC	29.25%
GEICAM/2006-03 (15)	2012	2	TNBC	EC-DCb	29.79%	EC-D	34.78%
GeparOcto (16)	2019	3	T1c-T4a-d TNBC and HER2+ BC	PMCb	51.72%	ddEPC	48.50%
GeparNuevo (17)	2019	2	T2-T4a-d TNBC	Durva(nab-P-ddEC)	53.40%	nab-P-ddEC	44.20%
I-SPY2 (18)	2020	2	high-risk stage II–III BC	PembroP-AC	67.80%	P-AC	21.35%
IMpassion031 (19)	2020	3	stage II–III TNBC	Atezo(nab-P-AC)	57.60%	nab-P-AC	41.10%

pCR, pathological complete response; A, doxorubicin; C, cyclophosphamide; Cb, carboplatin; D, docetaxel; E, epirubin; M, non-pegylated liposomal doxorubicin; P, paclitaxel; nab-P, nab-paclitaxel; dd, dose-dense; Durva, durvalumab; Pembro,pembrolizumab; Atezo, atezolizumab.

Altogether, a total of 1,647 patients were included from the final selected studies, of whom 845 received standard AT-based NACT and 802 received ICIs plus chemotherapy or platinum-based chemotherapy.

### Quality Assessment

**Figure 2:**
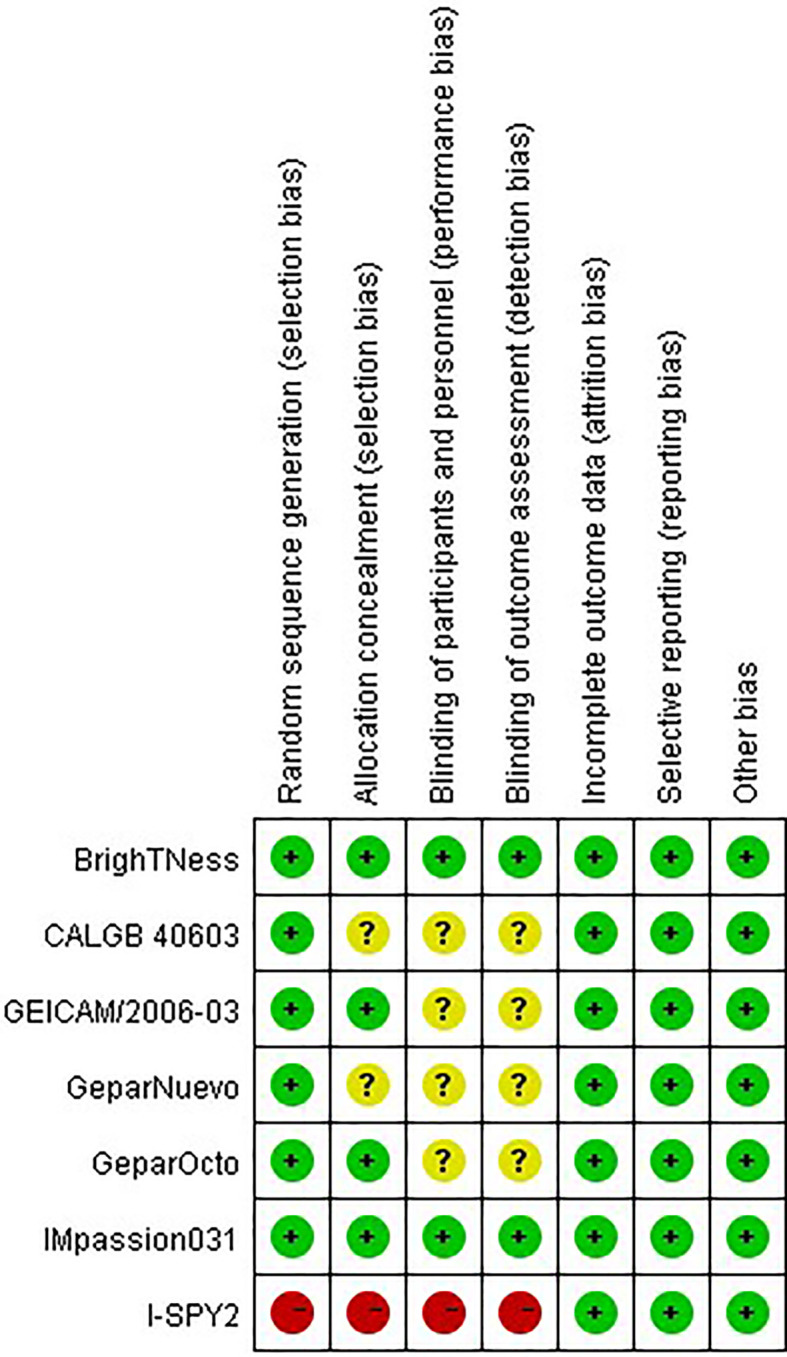
Quality assessment for risk of bias for the included randomized controlled trials.

**Figure 3:**
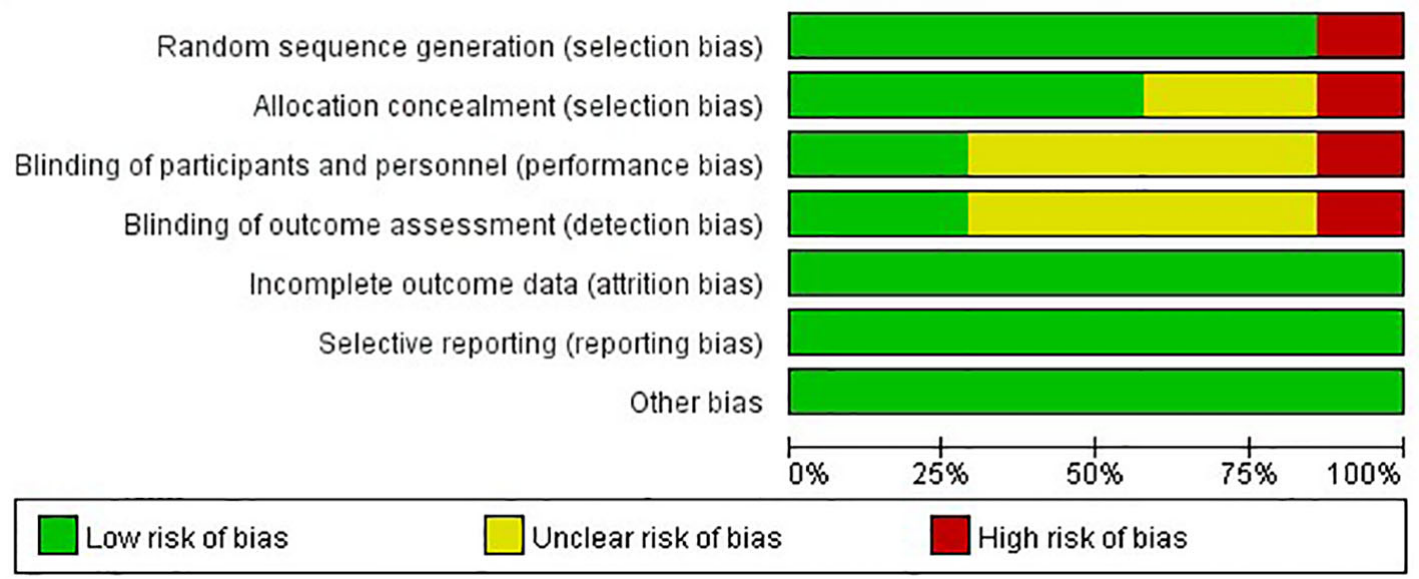
Risk of bias for the included randomized controlled trials.

The risks of bias of the included studies were appraised according to the Cochrane risk of bias tool (**Figure 2**). Six studies randomly allocated patients to the treatment arms, but only the I-SPY2 trial referred to the adaptive randomization method that may affect the results. Only two studies announced that all trial personnel and participants were masked to the assignment throughout the study course, while three studies did not mention the masking information. All studies had online registration information. Overall, these characteristics suggested moderate risks of study-design bias (**Figure 3**).

### pCR Rate

**Figure 4:**
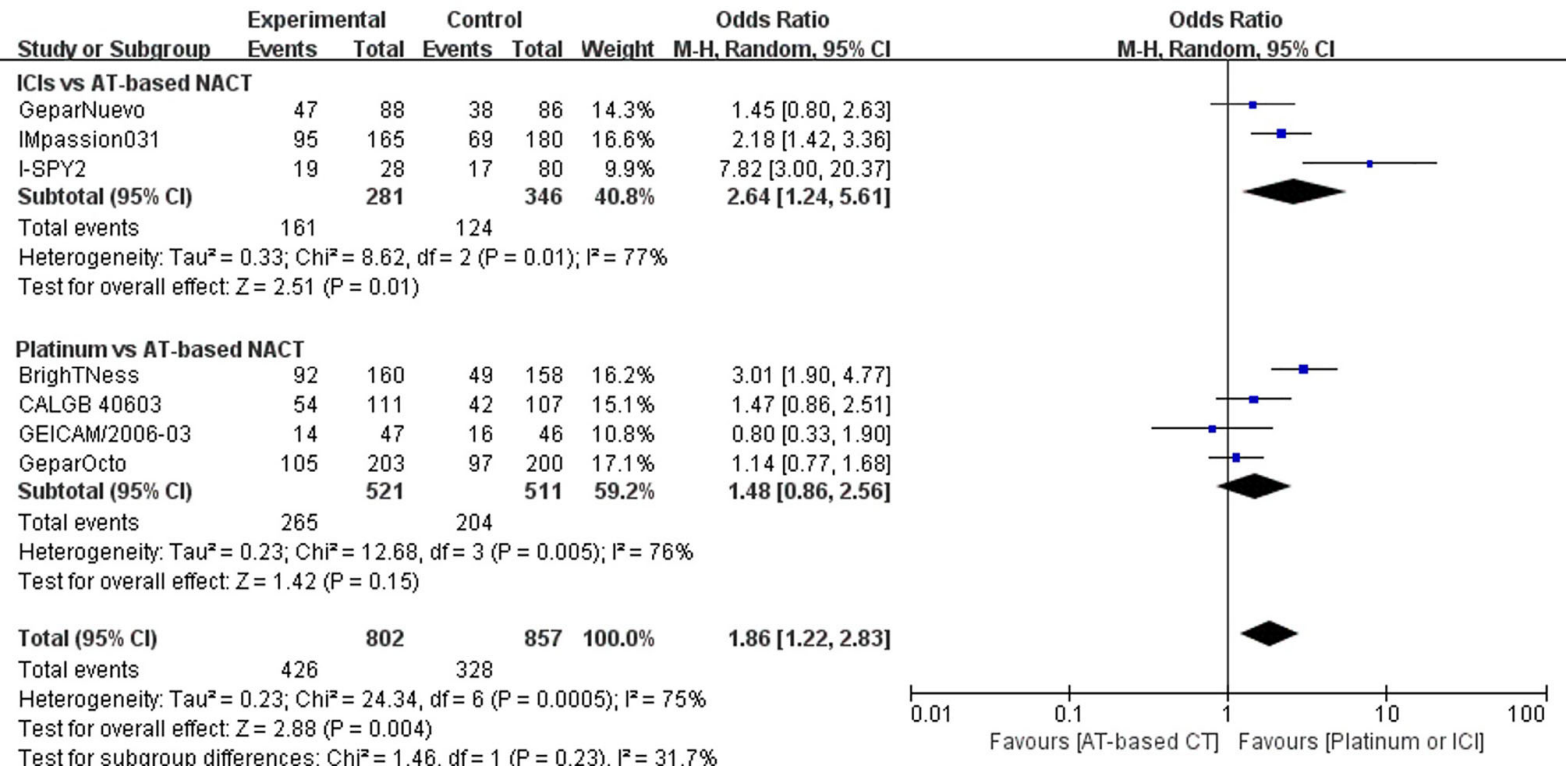
Forest plot showing pooled OR of pCR in patients receiving ICIs *vs* AT-based NACT and platinum-based *vs* AT-based NACT in early TNBC patients.

Altogether, four trials comparing platinum-based and AT-based NACT did not show a statistically significant improvement in pCR rate (P = 0.16); 265 out of 521 patients (50.86%) reached pCR in the experimental group, and 204 out of 511 patients (39.92%) reached pCR in the control group (OR, 1.48; 95%CI, 0.86–2.56). Trials have considerable heterogeneity (I^2^ = 77%) and evaluation with random-effects model was done (**Figure 4**).

A summary OR obtained with random effect models indicates that compared with AT-based NACT, the addition of ICIs significantly improved the pCR rates (P = 0.02) with 161 out of 281 (57.30%) reaching PCR in the experimental group and 124 out of 346 patients (35.84%) reaching pCR in the control group (OR, 2.64; 95%CI, 1.24–5.61). Trials have considerable heterogeneity (I^2^ = 76%), and evaluation with random effects model was done (**Figure 4**).

In the indirect comparison anchored in AT-based NACT (**Table 2**), ICIs plus chemotherapy demonstrated significant improvement in PCR rate *versus* platinum-based chemotherapy (p = 0.00445, OR, 1.78; 95%CI, 0.70–4.53)

**Table 2:** Results of indirect comparison.

	OR	95%CI	P
**pCR**	1.78	0.70–4.53	0.00445
pCR*	1.61	1.02–2.54	0.02199
**discontinuation related to AE**	0.46	0.26–0.82	0.00015
**grade 3-4 neutropenia**	0.2	0.03–1.56	<0.00001
**grade 3-4 anemia**	0.04	0.01–0.22	<0.00001
**grade 3-4 thrombocytopenia**	0.05	0.00–0.72	<0.00001

*Results excluding I-SPY2 and BrighTNess study.

### Sensitivity Analysis and Publication Bias

**Figure 5:**
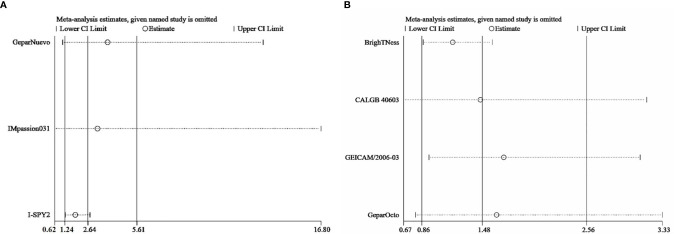
Sensitivity analysis comparing the incidence of pCR in patients receiving ICIs *vs* AT-based NACT **(A)** and platinum *vs* AT-based NACT **(B)**.

**Figure 6:**
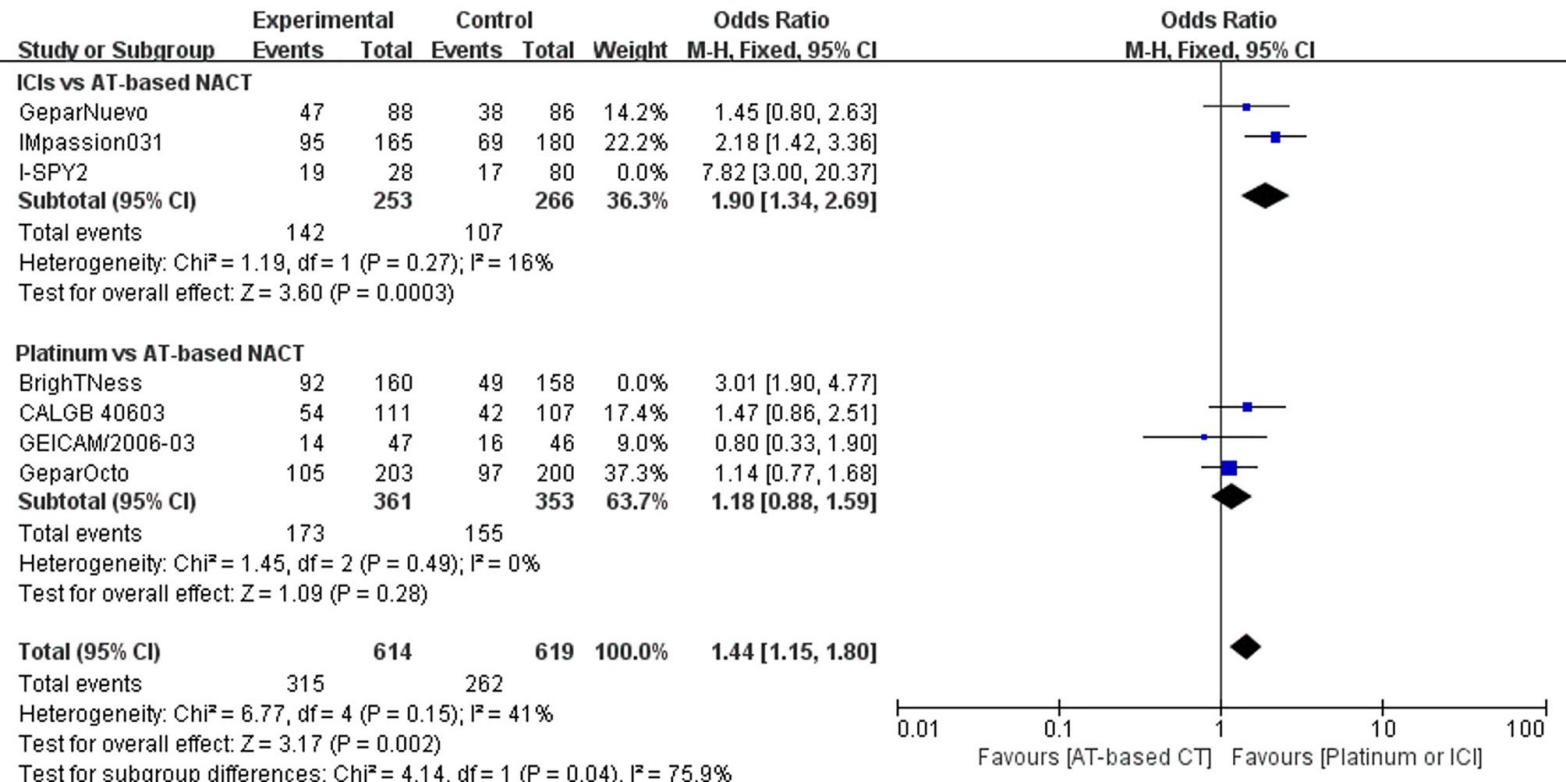
Forest plot showing pooled OR of pCR in patients receiving ICI *vs* AT-based NACT excluding I-SPY2 trial and in platinum *vs* AT-based NACT excluding BrighTNess trial.

**Figure 7:**
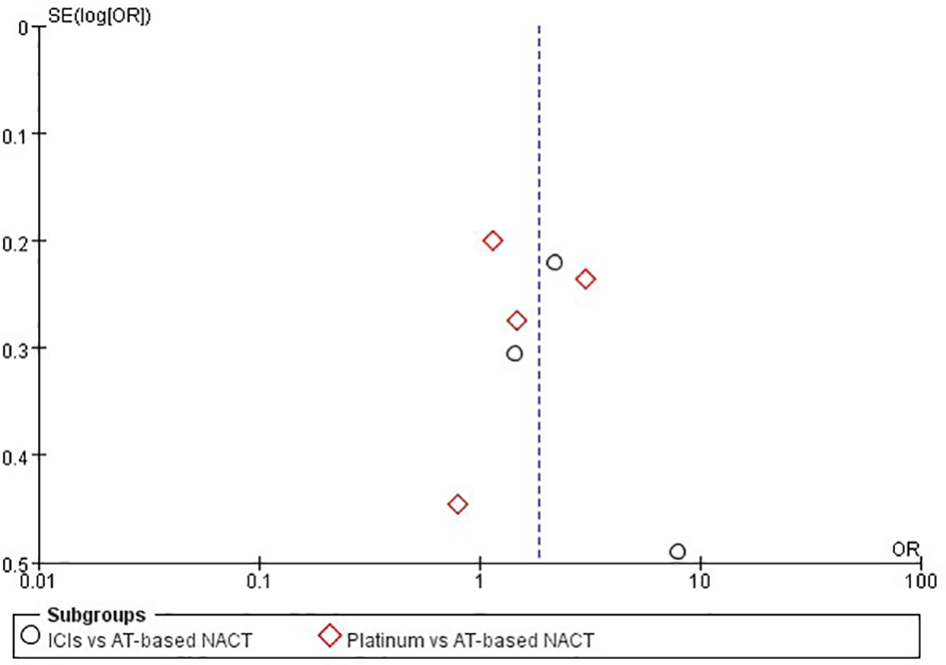
Funnel plot assessment of publication bias for pCR in patients receiving ICIs *vs* AT-based NACT and platinum-based *vs* AT-based NACT in early TNBC patients.

Sensitivity analysis was performed by excluding each study in both direct comparisons. The results showed stable pooled OR estimates in pCR rates (**Figure 5**). Notably, when omitting I-SPY2 study in ICIs *versus* AT-based NACT comparison, heterogeneity was significantly decreased (I^2^ = 77 to I^2^ = 16, **Figure 6**). Similarly, in platinum *versus* AT-based NACT comparison, heterogeneity was eliminated when excluding BrighTNess study (I^2^ = 76 to I^2^ = 0, **Figure 6**). When simultaneously excluding the two studies and evaluating with fixed-effect model, the result from the indirect comparison demonstrated that ICIs plus chemotherapy improved the pCR rate than platinum-based chemotherapy (OR, 1.61; 95%CI, 1.02–2.54) with statistical significance (P = 0.02), which was consistent with the primary comparison. No publication bias was detected by funnel plot (**Figure 7**).

### Subgroup Analysis of pCR Based on Lymph Node Status

Subgroup analysis stratified by regional LN status was available in only two trials, namely BrighTNess and Impassion031; the former compared platinum-based and AT-based NACT, while the latter studied the efficacy of the addition of ICIs to standard chemotherapy. The lack of enough stratified information and subgroup outcome in most trials precluded the calculation of a pooled estimate.

In the LN negative subgroup, the BrighTNess trial demonstrated that the addition of platinum statistically improved the pCR rate than AT-based NACT alone (risk difference, 29.1; 95%CI 15.0–43.3). However, in the Impassion031 trial, LN negative patients did not benefit from the addition of ICIs to chemotherapy (rate difference, 9; 95%CI −5 to 3). As to the LN positive subgroup, the two trials mentioned above indicated that platinum-based chemotherapy or the addition of ICIs can improve the pCR rate than standard AT-based NACT in the neoadjuvant setting in TNBC with statistical significance.

### Event-Free Survival

Only two of the included studies mentioned event-free survival (EFS), and their results were immature. In the Impassion031 trial, hazard ratio (HR) for EFS in the ICI group *versus* the chemotherapy group was 0.76 (95% CI 0.40–1.44) after 20.6 months of follow-up. In the I-SPY2 trial, after 2.8–3.5 years of median follow-up (depending on the arm), HR for EFS in the ICI arm *versus* the chemotherapy arm was 0.60.

### Safety Profile

#### Discontinuance Related to Adverse Effects

**Figure 8:**
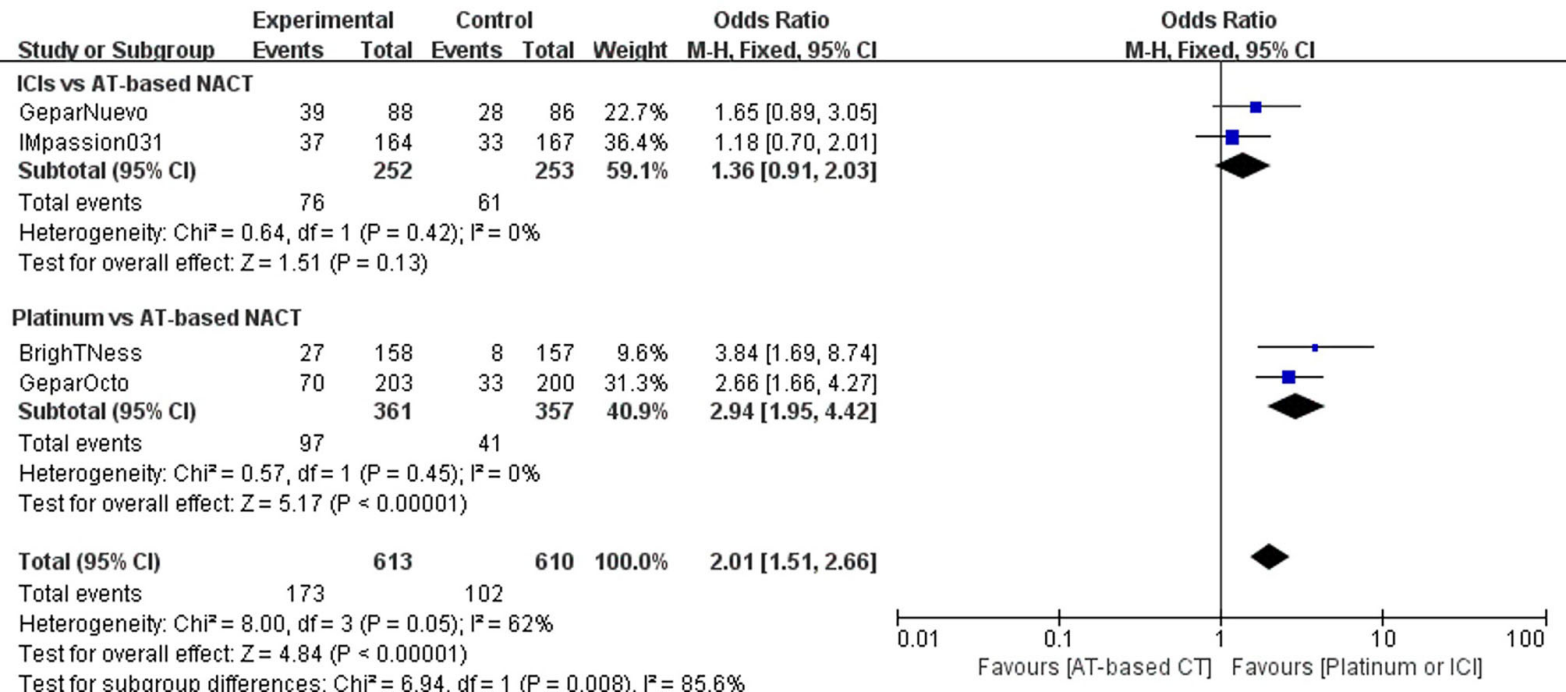
Forest plot showing pooled incidence of discontinuation due to AE in patients receiving ICIs *vs* AT-based NACT and platinum-based *vs* AT-based NACT in early TNBC patients.

Four trials reported discontinuations due to serious adverse effect during the treatment course. The addition of ICIs to AT-based NACT slightly decreased the adherence of the treatment (OR, 1.36; 95%CI, 0.91–2.03) with no statistical significance (P = 0.13). However, more patients in the platinum group required treatment discontinuation due to severe adverse effects than those given AT-based NACT (OR, 2.94; 95%CI, 1.95–.42) with statistical significance (P < 0.00001). Both trials have no heterogeneity (I^2^ = 0), and a fixed-effects model was employed (**Figure 8**).

In the indirect comparison between ICIs and platinum drugs, treatment terminations related to AEs occurred much less frequently in patients receiving ICIs plus chemotherapy than those receiving platinum-based chemotherapy(OR, 0.46; 95%CI, 0.26–0.82). The difference was statistically significant (P = 0.00015).

#### Hematological Effect

**Figure 9:**
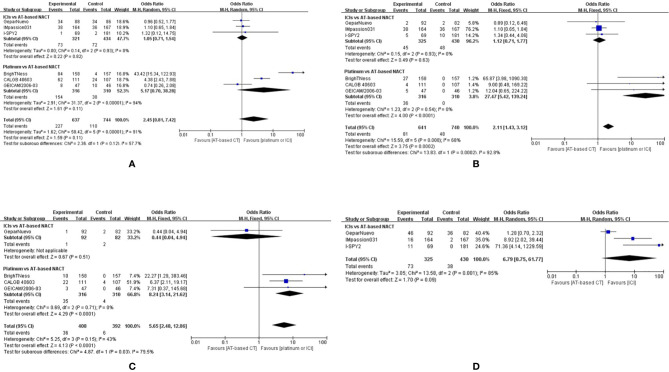
Forest plot showing pooled incidence of grade 3–4 neutropenia **(A)**, anemia **(B)**, thrombocytopenia **(C)** in patients receiving ICIs *vs* AT-based NACT and platinum-based *vs* AT-based NACT in early TNBC patients, all grades of thyroid dysfunction **(D)** in patients receiving ICIs *vs* AT-based NACT.

All seven studies included reported Grade 3–4 hematological adverse events (neutropenia, anemia, and thrombocytopenia), but the GeparOcto trial did not report adverse events for each treating arm separately. Grade 3–4 neutropenias occurred more commonly in the group given platinum than AT-based NACT (OR, 5.17; 95%CI, 0.70–38.28), although the difference was not statistically significant (P = 0.11). While the addition of ICIs to chemotherapy did not improve the rate of neutropenia (OR, 1.05; 95%CI, 0.71–1.54 P=0.82) (**Figure 9A**).

The indirect comparison demonstrated that compared with platinum-based chemotherapy, ICIs significantly reduced grade 3–4 neutropenia (OR, 0.20; 95%CI, 0.03–1.56, P < 0.0001). Similarly, patients given platinum-based chemotherapy have a higher chance of developing grade 3-4 anemia and thrombocytopenia compared to those who were given ICIs plus chemotherapy (**Figures 9B, C**).

#### Immune-Related Effect

Three studies comparing ICIs with AT-based NACT reported immune-related adverse effects. The most common immune-related effect is thyroid dysfunction, including hypothyroidism and hyperthyroidism. Altogether, 73 out of 325 patients develop thyroid dysfunction given ICIs plus chemotherapy, compared to 38 out of 430 patients in AT-based NACT group (**Figure 9D**). Thyroid dysfunction was more common in the ICI group (OR, 6.79; 95%CI, 0.75–61.77), but no statistical significance was found (P = 0.09). A random-effect model was employed due to the considerable heterogeneity (I^2^ = 85%). Among all three trials mentioned above, only one patient treated with pembrolizumab in the I-SPY2 trial developed grade 4 hypothyroidism, while all the other patients experienced mild thyroid dysfunction.

## Discussion

Triple-negative breast cancer is often associated with aggressive clinical behavior and early relapse, often affecting young women with a harsh impact on personal and social life. In the preoperative setting, a large number of studies have been conducted to discover the most efficient treatment to improve the clinical outcome. Platinum-based chemotherapy is recommended in multiple guidelines rather than conventional AT-based NACT for improvement of the PCR rate and disease-free survival. Some research studies also demonstrated increased rate of hematological adverse effects when patients were given platinum drugs that may lead to dose reduction or even discontinuation.

The recent studies on ICIs have offered more therapeutic approaches for TNBC, with FDA accelerating approval of atezolizumab for advanced TNBC based on the Impassion130 study (20, 21). A few phase 2 and 3 studies also offered promising results in the neoadjuvant setting. However, in the NEOTRIP trial (22), when compared to platinum-based chemotherapy, the addition of ICIs did not show statistical benefit in pCR rate, contrary to the result in the KEYNOTE-522 trial (23), which were the only two studies simultaneously covering platinum and ICIs.

Our study aimed to compare the treatment efficacy and safety between platinum drugs and ICIs indirectly mediated by AT-based chemotherapy given the absence of a head-to-head randomized controlled trial of these two drugs in TNBC patients. Results of this study demonstrated that the pCR rate is higher in the neoadjuvant setting when patients are given ICIs plus standard chemotherapy rather than platinum-based chemotherapy, with a statistically significant improvement in the intention-to-treat population. The considerable heterogeneity was mainly indicated by I-SPY2 study in ICI *versus* AT-based NACT comparison and BrighTNess study in platinum *versus* AT-based NACT comparison, which could be a result of different randomization methods. The adaptive randomization method was employed in I-SPY2 study, while permuted block randomization was employed in BrighTNess trial that could probably increase selection bias (24). When excluding these two studies simultaneously, the result from indirect comparison showed advantages in pCR in the ICI group consistently with the primary result, which increased the reliability of our study. However, the small number of included trials decreased the power of the results and was the main limitation of our study.

In patients with positive regional LN, the addition of either platinum or ICI can improve the pCR rate than AT-based NACT alone, while patients with negative LN seemed to only benefit from platinum drugs. However, the relationship between LN status and the response to different treatment regimens remains controversial since only two trials reported subgroup pCR information.

Two trials reporting EFS data, namely the I-SPY2 and Impassion031, were both not powered to detect an EFS increase with the addition of ICIs. Notably, in the recent KEYNOTE-522 trial (23) investigating ICI plus platinum-based chemotherapy *versus* platinum-based chemotherapy alone, with an 18-month follow-up, the event-free rate was 91.3% (95% CI, 88.8 to 93.3) in the experimental group and 85.3% (95% CI, 80.3 to 89.1) in the control group; the median was not reached in either group. The result favored the ICI group (HR, 0.63; 95% CI, 0.43 to 0.93), which suggested a potential benefit in EFS when patients were additionally given ICIs to platinum drugs. However, the improvement in pCR rates is possibly a result of down-staging of low-volume residual disease, which is not known to translate to a lower recurrence rate. Additional follow-up analyses are needed to investigate the relationship between pCR rate and survival outcome in these two treatments.

In terms of toxicity, no significant increase was observed for grade 3–4 AEs and treatment terminations in patients receiving ICI than AT-based NACT which had an extra advantage over platinum-based chemotherapy, with much less severe hematology AEs and treatment withdrawal. On the other hand, giving ICI treatment could result in a higher rate of thyroid dysfunction, which was not statistically significant, and only a few patients developed grade 3–4 immune-related AEs. The immune-related AEs, although relatively minor, may be a concern for a curative intention. The significantly increased and serious hematological toxicity of platinum drugs suggests a more careful choice for certain patients and should be balanced with its expected benefit.

Previous studies suggested that platinum drugs were particularly active in the treatment of breast cancer that develops in women with germline BRCA mutations. Several studies proved that platinum could lead to a higher response rate for metastatic TNBC with BRCA mutation (25). However, in the preoperative setting, subgroup analyses from the BrighTNess trial reported less improvements in pCR rate with platinum in patients with germline BRCA mutations compared with those with wild-type disease. A recent meta-analysis also suggested that the addition of platinum to neoadjuvant chemotherapy did not significantly improve the pCR rate for patients with BRCA mutations (26). The predictive value of homologous recombination deficiency (HRD) biomarker in clinical response to platinum-containing neoadjuvant treatment was also studied, and contradictory results were reported (27, 28).

The PD(L)-1 status, on the other hand, was proved to be associated with the clinical response to ICIs in the recent meta-analysis (29), while a non-significant trend of pCR improvement was also observed in PDL-1 negative subgroup, indicating the potential efficacy of immune therapy in PDL-1 negative population. Noting the limited and unclear results on those clinical biomarkers, further research studies are needed for optimization of individual treatment selection between platinum drugs and ICIs for patients with TNBC.

The limitation of our study could also be caused by the interaction and compatibility between the treatment agents. In the NeoTRIP trial (22), which was excluded in this meta-analysis, it can be observed that the addition of ICI to nab-paclitaxel and carboplatin did not significantly increase the rate of pCR in patients with TNBC, while doxorubicin induction could lead to an increase in clinical response to immune therapy in metastatic TNBC according to the result of TONIC trial (30). In platinum-based chemotherapy, the NeoSTOP study (31) indicated that six cycles of docetaxel and carboplatin achieved encouraging pCR and survival rates similar to paclitaxel plus carboplatin followed by doxorubicin and cyclophosphamide, yet with lower treatment toxicity. The compatibility of ICIs and platinum drugs should be considered for chemotherapy de-escalation strategies.

In general, our study provided an indirect comparison of platinum-containing treatment and ICIs plus chemotherapy in early TNBC using conventional AT-based chemotherapy as a common anchor. ICIs plus chemotherapy significantly improved pCR rates and decreased treatment toxicity than platinum-based chemotherapy in the general population. More clinical outcomes on survival data and subgroup information are required for optimal treatment choice, along with reliable biomarkers to identify potential patients who would benefit from specific treatment agent. Concerning the increased toxicity and inconclusive survival data, platinum drugs might not be suitable as a routine treatment for TNBC, even for those with BRCA mutations, while ICIs need to be further explored to confirm its efficacy and safety in neoadjuvant setting.

## Data Availability Statement

All data generated or analyzed during this study are included in this published article. Further inquiries can be directed to the corresponding authors.

## Author Contributions

Conceptualization, JL and QH. Data curation, QH and YP. Writing—original draft, QH. Writing—review and editing, JS and JL. All authors contributed to the article and approved the submitted version.

## Conflict of Interest

The authors declare that the research was conducted in the absence of any commercial or financial relationships that could be construed as a potential conflict of interest.
